# Salivary Ceruloplasmin Ferroxidase & Oxidase Activities in Celiac Patients

**Published:** 2012-09

**Authors:** Hathama R. Hasan, Jasim M. Ghadhban, Zahraa I. Abudal Kadhum

**Affiliations:** 1 *Department of Chemistry, College of Science, Baghdad University, Baghdad, Iraq;*; 2 *Head of GIT Center, Ministry of Health, Baghdad, Iraq*

**Keywords:** saliva, ceruloplasmin, celiac disease, mucosal histopathological damage, electrophoresis

## Abstract

The aim of the current study was to evaluate salivary ferroxidase ceruloplasmin activities in celiac patients with different histopathological severity. This study included 75 celiac patients with different mean age (18.68 ± 11.13) year, who had positive screen for celiac antibodies, and who had gastrointestinal symptoms. In order to simplify the comparison with the healthy control group, celiac patients were divided into two groups according to their histopathological severity: severe (marsh IIIa, b, c) & less severe (marsh 0, I). All these patients have been evaluating for salivary ceruloplasmin (Cp) concentration and Cp ferroxidase activities. To confirm the presence of the enzymatic activity of this protein, polyacrylamide gel electrophoresis was carried out and then stained for Cp ferroxidase, as well as for Cp oxidase activity. Furthermore, the concentrations of salivary total protein, albumin, and globulin were measured in the studied groups. A significant increase (*p*<0.05) in salivary concentration of ceruloplasmin was found in all above mentioned patients groups in comparison to that of the control group, except for total villous atrophy (marsh IIIc) patients subgroup. Salivary Cp ferroxidase activity revealed statistically significant decrease among the patient groups as well as between them and the control group. The result of salivary total protein and globulin showed presence a significant increase (*p*<0.05) in comparison to that of the control group. Meanwhile albumin levels was found to increase non-significantly (*p*=0.186).

## INTRODUCTION

The most commonly used laboratory diagnostic procedures involve the analyses of the cellular and chemical constituents of blood. Other biologic fluids are utilized for the diagnosis of disease, and saliva offers some distinctive advantages. Diagnosis of disease via the analysis of saliva is potentially valuable for children and older adults, since collection of the fluid is associated with fewer compliance problems as compared with the collection of blood. Whole saliva can be collected non-invasively and by individuals with limited training, Furthermore no special equipment is needed for collection of this fluid. Kaufman and Lamster in 2002 (1) suggested that the analysis of saliva may provide a cost-effective approach for the screening of large populations. Saliva contains proteins, in concentration of approximately 3% of plasma protein level (2), and can be informative for disease detection and surveillance of oral health (3). This body fluid was used as a sample fluid in several pathological conditions, including celiac disease CD (4).

Celiac disease (CD), also known as a celiac sprue and gluten-sensitive entropathy, is an immune-mediated disorder that primarily affects the gastrointestinal tract (GI), characterized by chronic inflammation of the small intestinal mucosa causing villous atrophy, and malabsorption of nutrients after the ingestion of gluten and its related peptides, in the form of wheat, barley and rye cereals (5). The clinical manifestations of CD are changeable in nature and vary markedly with the age of the patient, the duration and extent of disease, and the presence of extra-intestinal pathological conditions (6-8). In addition, to the classical gastrointestinal form, a variety of other clinical manifestations of the disease has been described, including atypical and asymptomatic forms (9). The keystone treatment of CD patients is a lifelong elimination diet in which food products containing gluten are avoided (10, 11).

Several antioxidant maneuvers aim at modifying the oxidative status in CD patients like ceruloplasmin (Cp) (Ferroxidase; Iron (II): O2 oxidoreductase, EC 1.16.3.1); the major blue copper containing glycoprotein (12). It is a major enzymatic contributor to the antioxidant defense system of human plasma, it acts as an antioxidant by several mechanisms (13, 14) inhibiting iron-dependant lipid peroxidation and OH. formation from hydrogen peroxide by its ferroxidase activity (14, 15), reacting and scavenging H_2_O_2_ and superoxide anion, and inhibiting copper–induced lipid peroxidation by binding copper ions (13, 14). According to the literature over 90% of human copper is associated with Cp as a non dialyzable fraction and the remaining 5-10% of plasma copper is believed to be fairly loosely attached to albumin and histidine, and only traces of copper is present as free Cu++ (16). Because of its oxidase activity, Cp is also known as copper oxidase, this activity can be used for measurement of Cp. Ceruloplasmin performs its ferro-oxidase activity at the cell surface by binding of iron to transferrin which is the first step in the transformation of Fe++ to Fe+++.

Serum Cp level was reported to increase during sport, pregnancy and estrogen supplement, as well as in states such as infections, malignities, Hodgkin’s disease and cholangitis. While a decrease in this level was reported in malnutrition and malabsorbtion states, nephrotic syndrome, and primary biliar cirrhosis (15, 17).

The involvement of the oral biochemical changes in CD has not attracted much attention, although the mouth is part of the gastrointestinal system (18). To the best of our knowledge, so far few researches have been done dealing with salivary Cp level and its enzymatic activities in the patients (19, 20). Previously Cp oxidase and ferroxidase activities were studied in our laboratory in sera of celiac patients (21), therefore the current project aimed to study the changes in Cp level and its enzymatic activities in the saliva of patients with CD at different stages of the disease in comparison with that of the healthy individuals, in an attempt to check the possibility of using saliva as an alternative biologic fluid to serum for diagnostic purposes.

## MATERIALS AND METHODS

### Inclusion criteria

A total of 75 cases with different chief complaints and presentation like chronic diarrhea, bloating, chronic abdominal pain and short stature or if they were positive for a CD- antibody screen were included in this study. These patients attended to the center of Gastroenterology and Hepatology, they were referred from different hospitals in Baghdad and other governorates in Iraq during the period of May 2010 to June 2011.

The age of these patients ranged from 2 year to 43 years, all patients were subjected to a personal interview using especially designed questionnaire format full history with detailed information (age, sex, symptoms, autoimmune diseases, gluten diet if intake).

The control group consisted of 46 apparently healthy individuals who matched in age and gender with patients, and had no history for any gastro-intestinal problem (from the friends and relatives), which refused to subject to Oesophago-gastro-duodenoscopy (OGD).

### Endoscopic Biopsy

A minimum of 3 biopsies were taken from different sites of the distal part of the duodenum, further examination of the duodenum, stomach and Oesophagus were performed. Histological analyses of the biopsies were carried out by two blinded expert pathologists while withdrawing the scope, the biopsies were placed in 10% formalin in a ground glass tube (universal tube) (22).

The diagnosis of CD was based on the presence of villous atrophy (total, subtotal or partial) with increased intraepithelial lymphocyte (IEL) counts on initial endoscopic biopsies. These histological analyses were scored according to the Marsh 1992 classification (23) revised in 1997: [Marsh IIIa (partial villous atrophy), Marsh IIIb (subtotal villous atrophy), and Marsh IIIc (total villous atrophy)] (24).

### Collection and treatment of saliva samples

Unstimulated, whole, mixed-saliva samples of 1 to 5 ml. were collected on ice, under resting conditions in a quiet room between 8.0-9.0 A.M. Patients and healthy were asked to rinse their mouth with normal saline and to generate saliva in their mouth and to spit into a plastic container for 10 minutes. After collection, the saliva was immediately centrifuged at (2000 × g) for 10 minutes at 4°C. The resulting supernatant was stored frozen at –34°C in eppendrof tubes until assayed.

The conflicting results that were obtained when Cp ferroxidase activity was measured using crude saliva, led us to concentrate the crude saliva by using two different methods in order to obtain concentration of salivary proteins enough for Cp ferroxidase activity determination as well as for the electrophoresis (by the pure sucrose as a dehydrant and dry Sephadex G-25 medium.

### Determination of Cp concentration and its enzymatic activities

Holmberg-Laurell method has been used for Cp concentration estimation (12). Salivary oxidase activity of Cp was determined using the modified Rice method (25), while Cp ferroxidase activity was determined as described by Erel, 1998 (26).

### Determination of total Protein& albumin

The salivary total protein concentrations were determined by using modified Lowery method by Hartree (27). Bbovine serum albumin (BSA) was used as standard& the Protein concentration was expressed in g/l. while salivary albumin concentrations were estimated by the method employing bromocresol green as described by Doumas *et al*. 1971 (28).

### Polyacrylamide Gel-Electrophoresis (PAGE)

Continuous 7.5% polyacrylamide gel-electrophoresiswas performed using LKB Electrophoresis apparatus (LKB power supply 2197, LKB multiphor 2117 & multitemp 2209) (29) to separate proteins in saliva samples. While discontinuous Lammeli system of native polyacrylamide gel-eectrophoresis (4% stacking gel and 7.5% separating gel) was used for Cp ferroxidase activity determination using Pharmacia gel electrophoresis apparatus GE-2/4 LS.

The polyacrylamide gel was stained for proteins using Coomassie Brillant Blue G-250 (CBB) (30), Cp oxidase using PPD (P-phenylenediamine) as a substrate (31), and ferroxidase enzymatic activities using Fe (II) as a substrate (32, 33).

### Statistical Analysis

The data were analyzed by Duncan's multiple range test at (*p*<0.05) was accepted as statistically significant, and highly significant when (*p*<0.001), using the SPSS software. All the analyses were repeated three times.

## RESULTS

The mean ages of the patients included in the current study were 14.58 ± 9.77 year for more severe histopathological celiac group (marsh IIIa, b, c), 17.807 ± 11.707 year for non & less severe histopathological celiac group (marsh 0, I), and 15.80 ± 10.32 for the control group.

**Figure 1:**
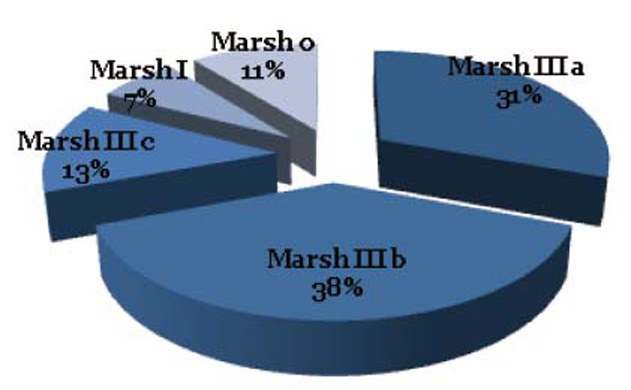
Marsh distribution among study groups.

The sex distribution of patients shows a statistical difference between the female (61%) and the male (39%). Meanwhile only 26.6% of all patients were in gluten free diet (GFD), it is worth to mention that most celiac patients included in the present study were found to be at stage III (82%), with the higher ratio of 38% in marsh IIIb, then 31% in marsh IIIa, and 13% in marsh IIIc; while the percentage of marsh I and 0 were 7% and 11% respectively (Fig. 1).

Table 1 showed the mean value of total protein in saliva samples, and revealed a significant increase (*p*=0.035) for the patients group in comparison to that of control group. The levels of salivary albumin showed non- significant increase (*p*>0.05), while salivary globulin indicated a significant increase (*p*=0.00).

**Table 1:** Mean laboratory values for salivary total protein, albumin and globulin with standard deviations in patients and control groups

	Celiac Patients				Control group	*P* value
	Marsh IIIa	Marsh IIIb	Marsh IIIc	Marsh 0, I		

Protein g/l (±SD)	2.34 ± 0.75	2.31 ± 0.71	2.94 ± 0.77	2.26 ± 0.88	2.00 ± 0.68	0.035
Alb. g/l (±SD)	0.33 ± 0.18	0.32 ± 0.15	0.28 ± 0.08	0.28 ± 0.19	0.28 ± 0.12	0.186
Glob. g/l (±SD)	2.03 ± 0.55	1.99 ± 0.61	1.79 ± 0.50	1.98 ± 0.40	1.72 ± 0.48	0.00

The results in Table 2 reveal a significant increase in the mean value of salivary Cp concentration of patients group in comparison to that of control group. A significant increase was observed in this protein concentration in partial and subtotal villous atrophy celiac patients (marsh IIIa, b), as well as in less histopathological mucosal change marsh (0, I) patients group, but non significant differences in total villous atrophy (marsh IIIc) celiac patients group, & as illustrated in Table 3.

**Table 2:** Mean value of salivary Cp concentration (mg/dl) in control and patient groups

Group	No.	Age (year) (Mean ± SD)	(Mean ± SD) mg/dl (Range)

Control	46	15.805 ± 10.324 (5-32)	0.738 ± 0.433 (0.5-0.995)
Patients marsh III	61	14.58 ± 9.772 (2-43)	a0.832 ± 0.27 (0.431-1.068)
Patients marsh 0, I	13	17.807 ± 11.707 (4.5-38)	a0.839 ± 0.222 (0.621-0.986)

a Signfficant difference in comparison to control at *P*<0.05.

**Table 3:** Mean value of salivary Cp concentration (mg/dl) in control and patient subgroups

Group	No.	Age (year) (Mean ± SD)	(Mean ± SD) mg/dl (Range)

Control	46	15.805 ± 10.324 (5-32)	0.738 ± 0.433 (0.5-0.995)
Patients marsh IIIa	23	13.956 ± 9.479 (2-43)	a0.893 ± 0.334 (0.621-1.142)
Patients marsh IIIb	28	14.144 ± 9.565 (2.5-33)	a0.857 ± 0.320 (0.431-1.068)
Patients marsh IIIc	10	19.400 ± 12.130 (5-39)	0.746 ± 0.269 (0.599-1.021)
Patients marsh 0, I	13	17.807 ± 11.707 (4-38)	a0.839 ± 0.222 (0.621-0.986)

a Significant difference in comparison to control at *P*<0.05.

**Figure 2:**
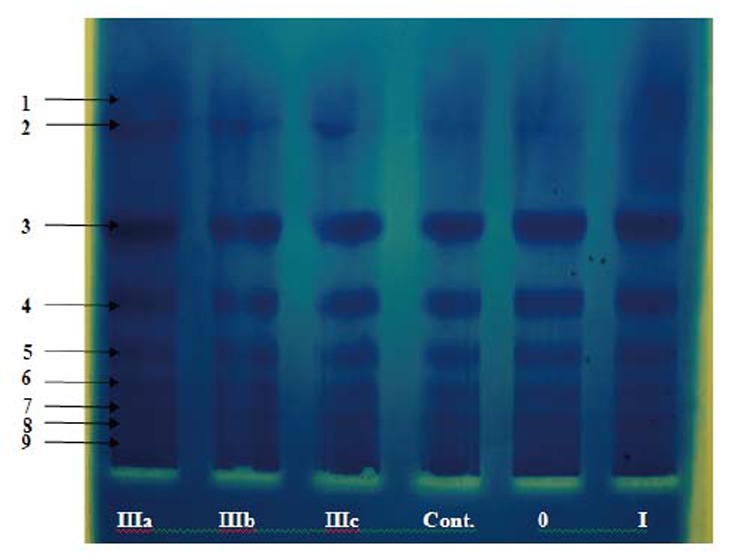
Conventional polyacrylamide gel electrophoresis (PAGE) 7.5%, using Tris- glycine buffer, pH 8.9 as electrode buffer. Electrophoresis was carried out for 3 hours at 4°C by using a constant current of 40 mA & voltage of 15 v/cm. The gel was stained for protein. Concentrated pooled saliva samples(with a protein concentration of 1.8 mg/ml ) were applied as follows: 1 p ooled c rude s aliva (marsh I IIa), 2 p ooled c rude saliva(r IIIb), 3 pooled crude saliva (marsh IIIc), 4 pooled crude saliva (control), 5 pooled crude saliva (mash 0), 6 pooled crude saliva (marsh I).

In order to investigate the differences in total protein pattern of the studied groups, a conventional polyacrylamide gel electrophoresis (PAGE) was carried out. The result is shown in Figure 2 & from the electrozymogram in the figure, differences among the studied groups in proteins profiles using CBB-G250 are clear. The salivary proteins were separated into distinct bands: albumin (band 3), α_1_- and α_2_-globulins (band 4 and 5), β_1_-and β_2_-globulins (band 6 and 7) and γ-globulins (band 8 and 9 respectively), where albumin moves faster than gamma globulin in the electrical field. It is clear from the electrogram that there is a visible increase in the density in the γ-globulins bands associated with an increase in albumin particularly in less severe histopathological lamina propria changes (marsh 0, I) as well as in partial villous atrophy (marsh IIIa) celiac patient groups.

Table 4 shows the ferroxidase activity of salivary Cp and its specific activity, which were measured in the concentrated crude saliva of all studied groups & as illustrated in the method section. As it is obvious from these results, the activity and the specific activity decrease with the severity of the disease.

**Table 4:** Illustrates the salivary Cp ferroxidase activities (U/ml) and specific activities in control and patients groups

Groups	Cp Ferroxidase activity (U/ml)	Cp Ferroxidase specific activity (U/mg)

Control	0.212	0.040
Patients marsh IIIa	a0.0958	a0.0159
Patients marsh IIIb	a0.097	a0.0172
Patients marsh IIIc	a0.1667	a0.0267
Patients marsh 0, I	a0.1687	a0.0298

a Significant difference in comparison to control at *P*<0.05.

**Figure 3:**
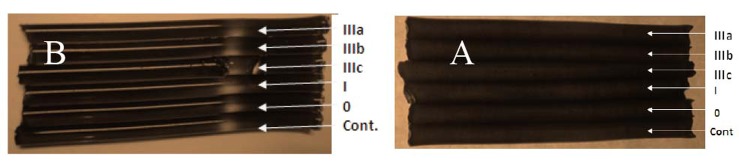
Discontinuous Polyacrylamide tubular Gel-Electrophoresis. (Lammeli system using 4% stacking gel and 7.5% separating gel) was carried out using Pharmacia Gel Electrophoresis Apparatus GE-2/4 LS. And Tris- glycine buffer, pH 8.2 as electrode buffer. Electrophoresis was carried out for 2.5 hours at 4°C with a constant current of 38 mA & a voltage of 15 v/cm. The gel was stained for CP ferroxidase activity. The applied concentrated samples (4.2 mg/ml) were as follows from up to down: pooled crude saliva (marsh IIIa), pooled crude saliva (marsh IIIb), pooled crude saliva (marsh IIIc), pooled crude saliva (marsh I), pooled crude saliva (marsh 0), and pooled crude saliva (control). (a) In the presence of sodium azide and (b) in the absence of sodium azide.

**Figure 4:**
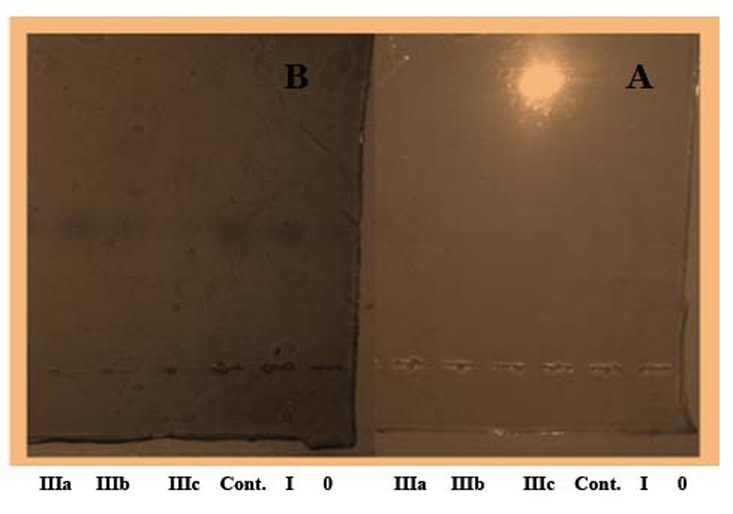
Conventional polyacrylamide slab gel electrophoresis (PAGE) 7.5%, using Tris- glycine buffer, pH 8.9 as electrode buffer. Electrophoresis was carried out for 3 hours at 4°C by using a constant current of 40 mA & voltage of 15 v/cm. The gel was stained for Cp oxidase activity. The applied samples (1.8 mg/ml) were as follows: 1- pooled crude saliva (control), 2 - pooled crude saliva (marsh IIIa), 3 - pooled crude saliva (marsh IIIb), 4 - pooled crude saliva (marsh IIIc), 5- pooled crude saliva (marsh 0), 6- pooled crude saliva (marsh I). (a) In the presence of sodium azide and (b) in the absence of sodium azide

In order to confirm that Cp in the saliva of the studied groups have the ferroxidase and oxidase activities, the polyacrylamide gel were stained, using PPD (13.8 mM) (Rice, 1962), and Fe (II) as substrates respectively, in the presence and absence of 1 mM sodium azide following Topham and Frieden method 1970, and the results are presented in Figures (3a and b) and (4a and b).

## DISCUSSION

In the current study, Salivary total protein levels were found to markedly increase (*p*=0.035). This result agrees with Lenander-Lumikari *et al* results (4), and disagrees with Mina S, *et al* (34) who reported a significant reduction in the total protein levels of celiac patients when compared with the control group, they pointed out that such reduction in total protein attributed to that those CD patients, who follow a strict gluten-free diet, secrete lower levels of amylase, myeloperoxidase, IgA and IgM in stimulated saliva than the control groups (34). In the present study the percentage of patients who were in GFD were only 26.6%, therefore the observed increase may be explained as follows: saliva, in general, contains arrays of proteins that have distinct biological functions, most of them have antibacterial, antimicrobial, and antibodies properties that defend the oral environment against any noxious agents, and such proteins were reported to increase in case of inflammation and tumors (35, 36). Also it was reported that many serum-derived proteins transferred to the saliva during inflammation (1). Throughout this study albumin level showed a non significant increase (*p*=0.186) in celiac patients when compared with that of the control group (Table 1). This may be due to the role of albumin as one of the extracellular antioxidants where this protein constitutes up to 49% of total plasma antioxidant status. Meanwhile albumin acts as sacrificial antioxidant by inhibiting the generation of free radicals through an immediate attacks of albumin molecule itself, so the radical reaction continue on albumin surface and cause damage to albumin molecule (37, 38) such damage is probably biologically insignificant, due to that albumin is present in plasma in high concentration (39), This result disagrees with Lenander-Lumikari *et al* (4) who reported a highly significant difference for albumin content in paraffin stimulated whole saliva in celiac patients (4).

Salivary Cp concentration showed statistically significant increased levels for patients groups (Table 2). In our previous study on serum Cp concentration in celiac patients, a significant increase was measured in the studied groups in comparison to the controls (21). So the salivary results could be explained on the basis that many molecules including Cp are capable of penetrating the gingival tissue through the intercellular spaces of the junctional epithelium to the saliva (20). As far as this protein concentration is concerned in the patient subgroups (Table 3); non- significant increase was observed in sera and saliva samples for celiac subgroup (total villous atrophy marsh IIIc), this result could reflect the severity of the disease in this patient's subgroup.

Frieden and Osaki suggested the presence of another protein in normal human serum (feeroxidase II), that differs greatly from ceruloplasmin. One of the properties which distinguish non ceruloplasmin ferroxidase II from that of ceruloplasmin is that this ferroxidase activity is not inhibited in the presence of 1.0 mM azide, while that of cp does (40). Such inhibition of cp ferroxidase activity is due to that sodium azide binds copper in ceruloplasmin and inhibits (95%-99%) of it's both ferroxidase and oxidase activities, a property which is considered as an evidence for the presence of Cp activities (26, 41).

Therefore an experiment was carried out where the polyacrylamide gel was stained for ferroxidase activity in the presence and absence of 1 mM of sodium azide in order to distinguish between this activity of both proteins. As it is clear from the electrozymogram in Figures 3 and 4. one clear band was obtained without sodium azide, while no band was detected when the staining was carried out using sodium azide This result confirm the presence of cp & its enzymatic activities in the saliva of the current studied patients. It is obvious from the electrozymogram (Fig. 4); there are no differences in cp band between the control and the patients group, as well as among the different patients' subgroups. A result which is different from what Daoud, 2008 reported where she observed distinct differences in the salivary zymograms of stained gel for Cp oxidase activity in oral tumor patients.

## CONCLUSION

To our knowledge, no research has been found in literatures dealing with salivary Cp concentration, or its enzymatic activities in celiac patients up to now. Our present study highlights the relationship between this disease at its different stages and salivary [Cp] & it's different enzymatic activities; further study is carrying out in our laboratory to investigate this relationship more deeply.
